# Oscillatory dynamics serving visual selective attention during a Simon task

**DOI:** 10.1093/braincomms/fcad131

**Published:** 2023-04-20

**Authors:** Jake J Son, Yasra Arif, Mikki Schantell, Madelyn P Willett, Hallie J Johnson, Hannah J Okelberry, Christine M Embury, Tony W Wilson

**Affiliations:** Institute for Human Neuroscience, Boys Town National Research Hospital, Boys Town, NE 68010, USA; Interdisciplinary Graduate Program in Biomedical Sciences (IGPBS), College of Medicine, University of Nebraska Medical Center (UNMC), Omaha, NE 68198, USA; Institute for Human Neuroscience, Boys Town National Research Hospital, Boys Town, NE 68010, USA; Institute for Human Neuroscience, Boys Town National Research Hospital, Boys Town, NE 68010, USA; Interdisciplinary Graduate Program in Biomedical Sciences (IGPBS), College of Medicine, University of Nebraska Medical Center (UNMC), Omaha, NE 68198, USA; Institute for Human Neuroscience, Boys Town National Research Hospital, Boys Town, NE 68010, USA; Institute for Human Neuroscience, Boys Town National Research Hospital, Boys Town, NE 68010, USA; Institute for Human Neuroscience, Boys Town National Research Hospital, Boys Town, NE 68010, USA; Institute for Human Neuroscience, Boys Town National Research Hospital, Boys Town, NE 68010, USA; Institute for Human Neuroscience, Boys Town National Research Hospital, Boys Town, NE 68010, USA; Interdisciplinary Graduate Program in Biomedical Sciences (IGPBS), College of Medicine, University of Nebraska Medical Center (UNMC), Omaha, NE 68198, USA; Department of Pharmacology & Neuroscience, Creighton University, Omaha, NE 68178, USA

**Keywords:** magnetoencephalography, oscillations, selective attention

## Abstract

Selective attention is an important component of cognitive control and is essential for day-to-day functioning. The Simon task is a common test of visual selective attention that has been widely used to probe response selection, inhibition and cognitive control. However, to date, there is a dearth of literature that has focused on the oscillatory dynamics serving task performance in the selective attention component of this task. In this study, 32 healthy adults (mean age: 33.09 years, SD: 7.27 years) successfully completed a modified version of the Simon task during magnetoencephalography. All magnetoencephalographic data were pre-processed and transformed into the time–frequency domain. Significant oscillatory brain responses were imaged using a beamforming approach, and peak task-related neural activity was extracted to examine the temporal dynamics. Across both congruent and Simon conditions, our results indicated robust decreases in alpha (8–12 Hz) activity in the bilateral occipital regions and cuneus during task performance, while increases in theta (3–6 Hz) oscillatory activity were detected in regions of the dorsal frontoparietal attention network, including the dorsolateral prefrontal cortex, frontal eye fields and insula. Lastly, whole-brain condition-wise analyses showed Simon interference effects in the theta range in the superior parietal region and the alpha range in the posterior cingulate and inferior frontal cortices. These findings provide network-specific insights into the oscillatory dynamics serving visual selective attention.

## Teaser

Using magnetoencephalography and a modified Simon task to investigate the neural oscillatory dynamics serving selective attention, Son *et al.* report decreases in alpha (8–12 Hz) activity in primary visual regions and increases in theta (3–6 Hz) activity in regions of the dorsal frontoparietal attention network.

## Graphical Abstract

**Graphical Abstract:**
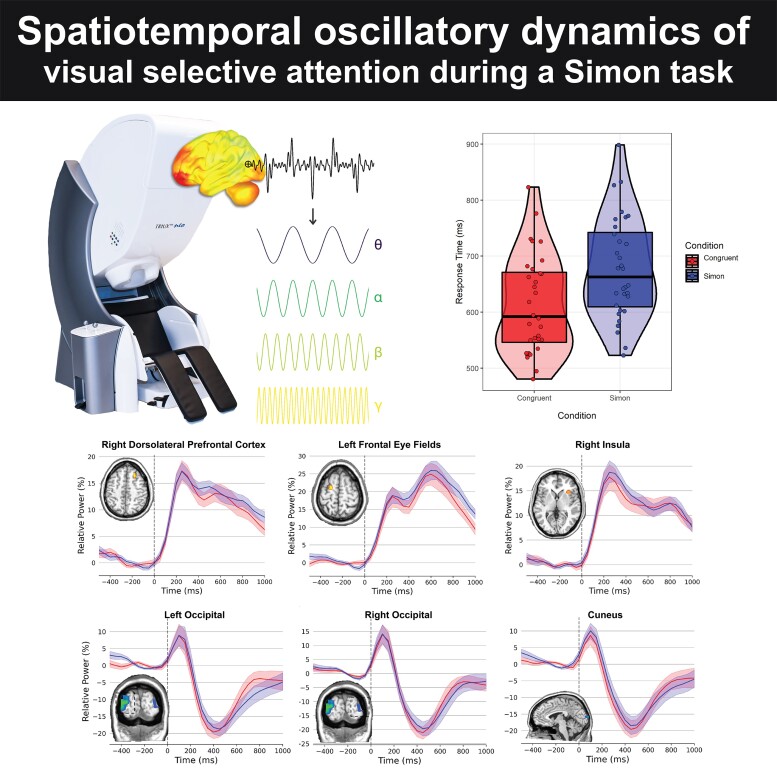

## Introduction

Cognitive control refers to a flexible construct that supports a wide range of mental operations by adaptively modulating information processing and behaviours to achieve specific goals in various contexts. Selective attention is a key mechanism of cognitive control and is closely monitored by a distributed network of brain regions including the frontal eye fields (FEF), dorsolateral prefrontal cortex (dlPFC) and superior portion of the parietal cortex, collectively referred to as the dorsal frontoparietal attention network.^1-4^ These regions exhibit concurrent activation during spatial selective attention and conflict processing when attentional resources are allocated towards relevant stimulus features while irrelevant information is inhibited.^5-9^ Visual selective attention, and subsequently cognitive control, are essential components of activities of daily living and alterations can impact individuals’ independence and quality of life. Alterations to visual selective attention, and by extension cognitive control processes and behaviours, have been identified in healthy development, trauma exposure, cannabis use and individuals with psychiatric and neurological disorders.^8-14^ Utilizing advanced neuroimaging techniques to better understand the neural bases of visual selective attention may improve our understanding of successful selective attention processes in healthy and clinical populations.

A number of selective attention tasks have commonly been employed to probe the neural responses of cognitive control at various steps of interference resolution, including conflict identification, response selection and response execution.^15-17^ In these tasks, behavioural changes either in response time or accuracy are impacted by the presence of distracting or irrelevant stimuli, which are inhibited via top-down selective attention processes. Substantial neuroimaging literature has explored the effects of cognitive interference on neural activity, documenting activation in the frontal, parietal and occipital cortices.^18-24^ One such classic neuroimaging task to probe cognitive control and selective attention is the Simon interference task,^17^ in which participants respond more quickly when the spatial features of the stimulus (task irrelevant) and response (task relevant) correspond (e.g. both the stimulus and response correspond to the left side of the screen). The conflict arises from incongruence between the stimulus and response features, and frequently leads to slowed reaction times as well as changes in neural activity during stimulus processing and decision-making. Despite this interference effect in response time, participants generally have high accuracy rates, and this is thought to reflect the successful utilization of visual selective attention.^25^

Selective attention is implicated in a distributed neural network with modulatory and target regions. Previous neuroimaging studies using adaptations of the Simon task during EEG and functional magnetic resonance imaging have identified such regions associated with selective attention in the anterior cingulate cortex, FEF, regions of the prefrontal cortex, parietal cortex and occipital cortex.^18,26-29^ In particular, recent EEG literature has examined the temporal dynamics of selective attention, identifying increased mid-frontal theta power,^30^ associations between alpha power and behavioural performance,^31^ as well as alpha frequency involvement in attentional shifts and spatial attention.^32-34^ Though the excellent temporal precision of EEG affords insights into the electrophysiological basis of selective attention, these findings are somewhat limited by spatial smearing that often prevents the separation of cortical sources.^35,36^ However, while functional magnetic resonance imaging and EEG are staple neuroimaging methods and afford either high spatial resolution (functional magnetic resonance imaging) or excellent temporal resolution (EEG), magnetoencephalography (MEG) is equipped with both high spatial and temporal precision and is relatively ideal for dissecting the neurophysiological mechanisms supporting cognitive control by directly measuring the neuro-magnetic activity that emanates from neuronal populations. Previous MEG studies using the Flanker task have identified neural regions associated with selective attention and phenotypic features that modulate such neural signatures, including the effect of cannabis use, healthy development, ageing and HIV.^8-10,13,14^ However, the Simon task literature is sparse relative to functional neuroimaging studies examining the Flanker and Stroop tasks. Furthermore, while the Flanker and Stroop tasks probe a combination of stimulus–stimulus and stimulus–response interference, the Simon task can be attributed solely to stimulus–response interference.^25^ Thus, examining the spatiotemporal dynamics specific to stimulus–response interference via the Simon task would expand upon prior imaging work using the Flanker and Stroop interference tasks and advance the field in terms of the specific impact of different types of cognitive interference.

As noted above, the neural dynamics serving selective attention and successful performance during the Simon task remain poorly understood. Previous MEG studies from our lab and others have identified spectrally specific rhythmic neural activity in healthy adults during the Flanker task and demonstrated modulations of these responses in clinical populations,^8,9,13,14,23^ but studies to date have yet to examine the spatiotemporal oscillatory dynamics serving Simon task performance in healthy adults. Thus, in the current study, we used a modified version of the Simon task to characterize the multispectral neural dynamics underlying cognitive interference.^23,24^ We hypothesized that participants would exhibit significant neural oscillations in dorsal frontoparietal attention regions associated with the successful completion of the Simon task.

## Materials and methods

### Participants

A total of 32 healthy adults (mean age: 33.09 years, 26 male) between the ages of 20 and 44 years old were recruited. Exclusion criteria included individuals with medical illness affecting central nervous system function, history of significant head trauma, current substance abuse, a formal diagnosis of neurological or psychiatric disorders (or any major medical conditions that may affect central nervous system function), or metal implants (e.g. retainer and pacemaker) that would negatively affect MEG data acquisition or be an MRI safety concern. All participants were screened for eligibility using a six-question screening form administered by trained research assistants. In addition, following enrolment, participants were also assessed using the NIH Toolbox Cognition Battery and were required to score in the normative range to be included in the study.^37-39^ The Institutional Review Board reviewed and approved this investigation, and written informed consent was obtained from each participant.

### Experimental paradigm and stimuli

**Figure 1:**
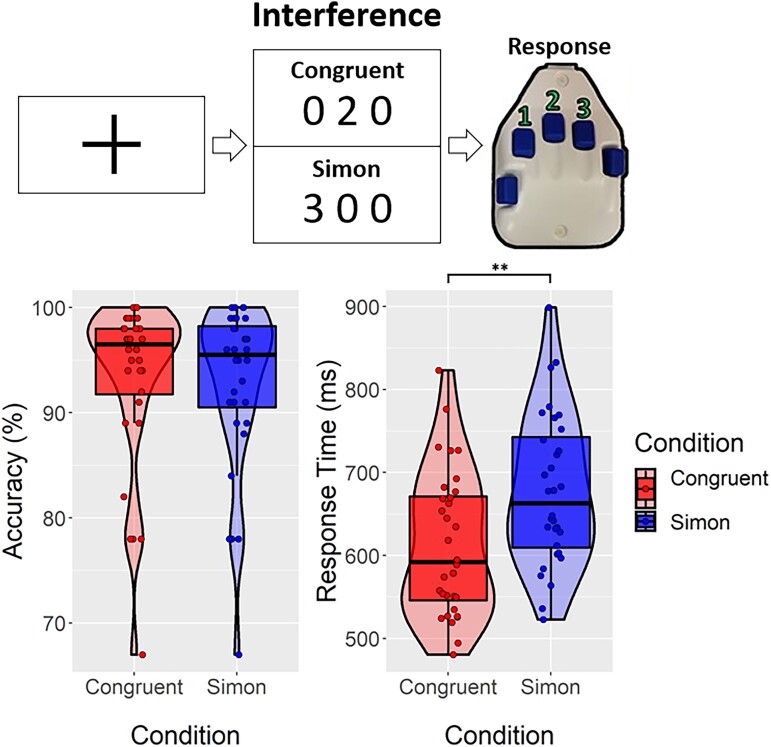
**Experimental paradigm and behavioural results.** (*Top*) Modified version of the classic Simon task. Participants (*N* = 32) were shown a centrally presented fixation cross for 2200 ± 200 ms, followed by three equally spaced, horizontally centred numeric stimuli (i.e. digits ranging from 0 to 3) for 1500 ms. For each trial, two of these numbers were identical (task irrelevant), and the third was different (task relevant). Participants were asked to indicate the ‘odd-number out’ by pressing the button corresponding to its numerical identity (i.e. index = 1, middle = 2 and ring = 3) and not its spatial location. Our primary goal was to examine the impact of the congruent (no interference; i.e. 1 0 0/0 2 0/0 0 3) versus Simon interference (e.g. 0 1 0/0 0 2/3 0 0) conditions. (*Bottom*) Results from the behavioural analyses, with accuracy (% correct) on the left and response time (ms) on the right with one data point per participant’s mean value. **P < 0.01, paired *t*-test. Participants responded significantly slower during the Simon compared with congruent trials

Each participant was seated in a magnetically shielded room and completed a modified version of the Simon task.^17^ Participants were shown a centrally presented fixation cross for 2200 ± 200 ms, followed by three equally spaced, horizontally centred numeric stimuli (i.e. digits ranging from 0 to 3) for 1500 ms. For each trial, two of these numbers were identical (task irrelevant), and the third was different (task relevant), and the placement and identity of these stimuli reflected the interference to be measured. For the purposes of this investigation, we examined the neutral or congruent (no interference; i.e. 1 0 0/0 2 0/0 0 3) condition and the Simon (e.g. 0 1 0/0 0 2/3 0 0) condition (Fig. 1). Participants were asked to indicate the ‘odd-number out’ by pressing the button corresponding to its numerical identity (i.e. index = 1, middle = 2 and ring = 3) and *not its spatial location*. To minimize bias by trial and response type, the experiment was pseudorandomized to prevent a given condition or response from being repeated more than twice in a row. Each participant completed 100 trials of each of the congruent and Simon trials for a total of 200 trials in this study. MATLAB was used to program custom visual stimuli (Mathworks, Inc.) using Psychophysics Toolbox V3^40^ and back projected onto a non-magnetic screen.

### Behavioural data analysis

For each participant, incorrect and no-response trials were removed prior to calculating the reaction time for each condition. Accuracy per condition was computed as the percentage of correct trials/total trials. On the single-participant level, a standard single-trial reaction time threshold of 3 SD was used to remove outlier trials. Mean reaction times were subsequently calculated for each participant and condition. Reaction time and accuracy data were then analysed using paired-samples *t*-test to identify conditional effects. All behavioural analyses were implemented in Python using the SciPy package.^41^

### MEG data acquisition

All MEG data were collected in a magnetically shielded room with a 306-sensor Elekta VectorView MEG system (Helsinki, Finland) sampled at 1 kHz with an acquisition bandwidth of 0.1–330 Hz. The system included 204 planar gradiometers and 102 magnetometers, but we focused on the planar gradiometers in this investigation. During data collection, study participants were monitored in real time via audio-visual stream from within the magnetically shielded room. Raw MEG data were noise corrected for head motion and extraneous noise using the signal-space separation method with a temporal extension.^42^

### Structural MRI processing and MEG coregistration

For a complete description of MRI processing and MEG coregistration, we refer the reader to our previous MEG work.^43-46^ In brief, prior to MEG acquisition, four head position indicator coils were attached to the participant’s head and localized in 3D space along with fiducials and the scalp surface using a 3D digitizer (Fastrak 3SF0002, Polhemus Navigator Sciences, Colchester, VT, USA). During data acquisition, each of the head position indicator coils was tracked in physical space relative to the MEG array, which was used to correct for motion. Each participant’s MEG data were co-registered with their T_1_-weighted structural MRI prior to source space analysis using BESA MRI (Version 2.0). These MRI data were transformed into standardized Talairach space. Following source analysis (i.e. beamforming), the same transformation that was done to the structural MRI volume was applied to each participant’s functional MEG images and spatially resampled.

### MEG pre-processing and time–frequency transformation

Cardiac and ocular artefacts were identified in the raw recordings using MEG sensors with the best representation for the specific source of interference (i.e. near the orbits for eye movements) and removed using signal-space projection, a method that assumes that magnetic field distributions generated by brain sources have spatial distributions sufficiently different from those generated by non-brain sources. Removal of these artefacts was accounted for during source reconstruction.^47^ The continuous MEG time series was epoched into 1500 ms segments, extending from 500 ms prior to visual stimulus presentation to 1000 ms after the stimulus. The baseline period was defined as the −500 to 0 ms window, while the active period ranged from 0 to 1000 ms. Epochs containing remaining artefacts were rejected per participant using a fixed threshold method, supplemented with a visual inspection.

Artefact-free epochs were transformed into the time–frequency domain using complex demodulation^48^ with a time–frequency window of 50 ms by 1 Hz and a bandwidth of 3–100 Hz. The resulting spectral power estimations per sensor were averaged across trials to generate mean spectral density plots. The data were then normalized per time–frequency bin using the respective bin’s baseline power, which was calculated as the mean power during the −500 to 0 ms time period. These normalized spectral power plots were then examined statistically to determine windows of interest for subsequent source analysis.

### MEG sensor-level statistics

For a complete description of MEG sensor-level statistics and source imaging, we refer the reader to our previous MEG work.^43-46,49^ Briefly, a two-stage procedure was followed to control for Type-1 error. Paired-sample *t*-tests relative to baseline were conducted for each data point prior to non-parametric cluster-based permutation testing to build a null distribution based on 10 000 permutations for temporally and/or spectrally neighbouring significant time–frequency bins (all *P* < 0.05). The significance level of the observed clusters (from Stage 1) was then tested directly using this distribution using the BESA Statistics 2.1 toolbox.^50,51^ The resulting time–frequency windows that contained statistically significant oscillatory events across all participants and conditions were subjected to a beamforming analysis.

### MEG source imaging and statistics

Neural responses were imaged using the dynamic imaging of coherent sources beamformer, which estimates the sensor-level cross-spectral density to derive the power spectra and coherence measures, which were used to calculate source power per voxel for the entire volume.^52^ Each task condition was imaged separately per participant for the statistically defined time–frequency bins (see the ‘Results’ section). For each location in the input voxel space, we computed noise-normalized source power per participant using the active (i.e. modified Simon task) and passive (i.e. baseline) periods of equal duration and bandwidth at a resolution of 4 mm isotropic.^53^ Such images are typically referred to as pseudo-t maps, with units (pseudo-t) that reflect noise-normalized power differences (i.e. active versus passive) per voxel.

### Statistical analysis

After imaging the neural responses, each time–frequency-specific neural response map was averaged across condition per participant and probed for task effects using whole-brain one-sample *t*-tests, with the resulting statistical map indicating which brain regions were generating the significant oscillatory brain responses observed in sensor space. We then examined the Simon effect using whole-brain paired-sample *t*-tests (congruent versus Simon) per time–frequency component. The resulting statistical maps illustrating the whole-brain Simon effect. MEG pre-processing and imaging used the BESA (version 7.0) software.

To visualize the temporal dynamics within the brain regions identified through the above statistical analyses, voxel time series (‘virtual sensors’) data corresponding to the peak voxel of each cluster were extracted. We used the sensor weighting matrix (from the forward solution) to estimate a time series for the specific peak coordinate (i.e. virtual sensor); we estimated one time series per cluster surviving the statistical analyses. Of note, the time series data corresponding to the voxels of interest were extracted per participant and condition.

## Results

### Behavioural results

The 32 participants performed well, with a mean accuracy of 93.19% (SD: 8.07%) in the congruent condition and 92.69% (SD: 8.01%) in the Simon condition. The mean reaction times for the congruent and Simon conditions were 613.14 ms (SD: 87.30) and 677.63 ms (SD: 90.79), respectively. While differences in accuracy were not significant, *t*(31) = 0.81, *P* = 0.422, differences in reaction time between conditions were significant, *t*(31) = 13.03, *P* < 0.001 (Fig. 1).

### MEG sensor-level results

**Figure 2:**
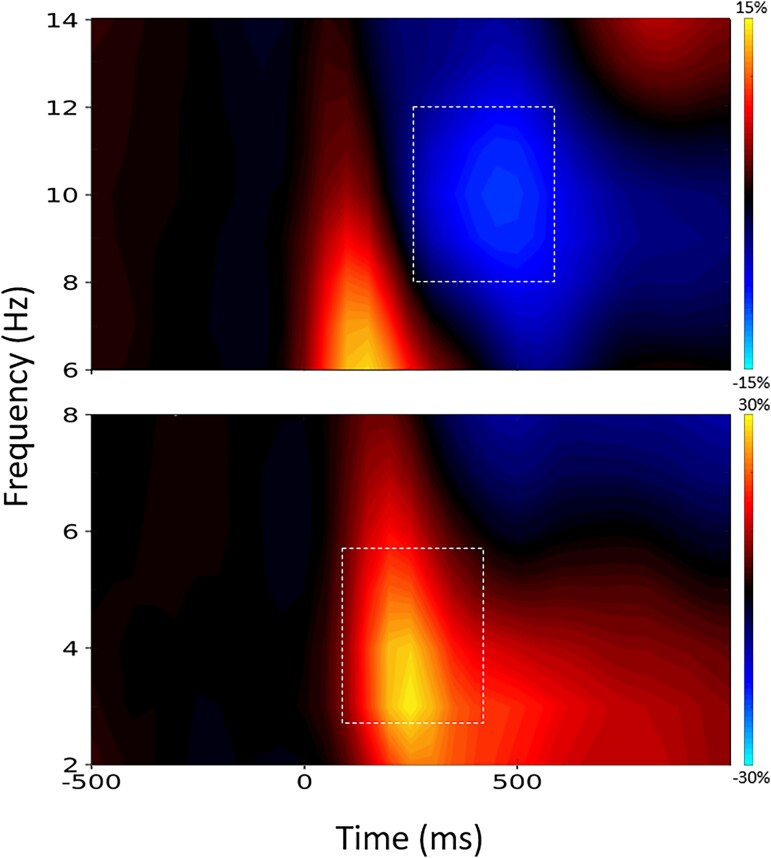
**Grand-averaged spectrograms during task performance.** The spectrograms display time–frequency representations averaged across all participants and both conditions. All signal power is represented as a percent difference in power from the baseline period, with the scale bar shown to the right. The *x*-axis represents time in ms units, with 0 ms as the stimulus onset, and the *y*-axis represents frequency in Hz. The time–frequency windows selected for beamforming are shown with dashed white boxes and were derived from nonparametric cluster-based permutation testing of the MEG sensor level data (*P* < 0.05). Each spectrogram represents grand-averaged data from one gradiometer sensor that was representative of the neural responses seen in multiple sensors near the peak response

Following artefact rejection, an average of 89.16 (SD: 7.41) trials remained in the congruent condition and 88.47 (SD: 8.08) trials remained in the Simon condition. The number of trials per condition did not statistically differ (*P* = 0.42). To derive the time–frequency bins for beamforming analyses, sensor-level spectrograms were probed using non-parametric permutation testing (see the ‘Materials and methods’ section). These analyses revealed clusters of sustained decreases (i.e. desynchronization) in the alpha band (8–12 Hz) activity that began 250 ms after stimulus onset and continued until 600 ms (*P* < 0.05, corrected; Fig. 2). In addition, there was increased activity in the theta range (3–6 Hz) that began 150 ms after stimulus onset and was sustained through 500 ms (*P* < 0.05). Note that these time windows were intentionally limited to 600 ms (i.e. prior to participants completing the trial) to capture attention processes during Simon performance. To evaluate the dynamics and determine the precise brain regions generating the oscillatory responses observed at the sensor level, each time window was imaged using a baseline period of equal bandwidth and duration (−350 to 0 ms), and then compared statistically for differences by condition.

### MEG beamforming and sensor-level results

#### Task effects

**Figure 3:**
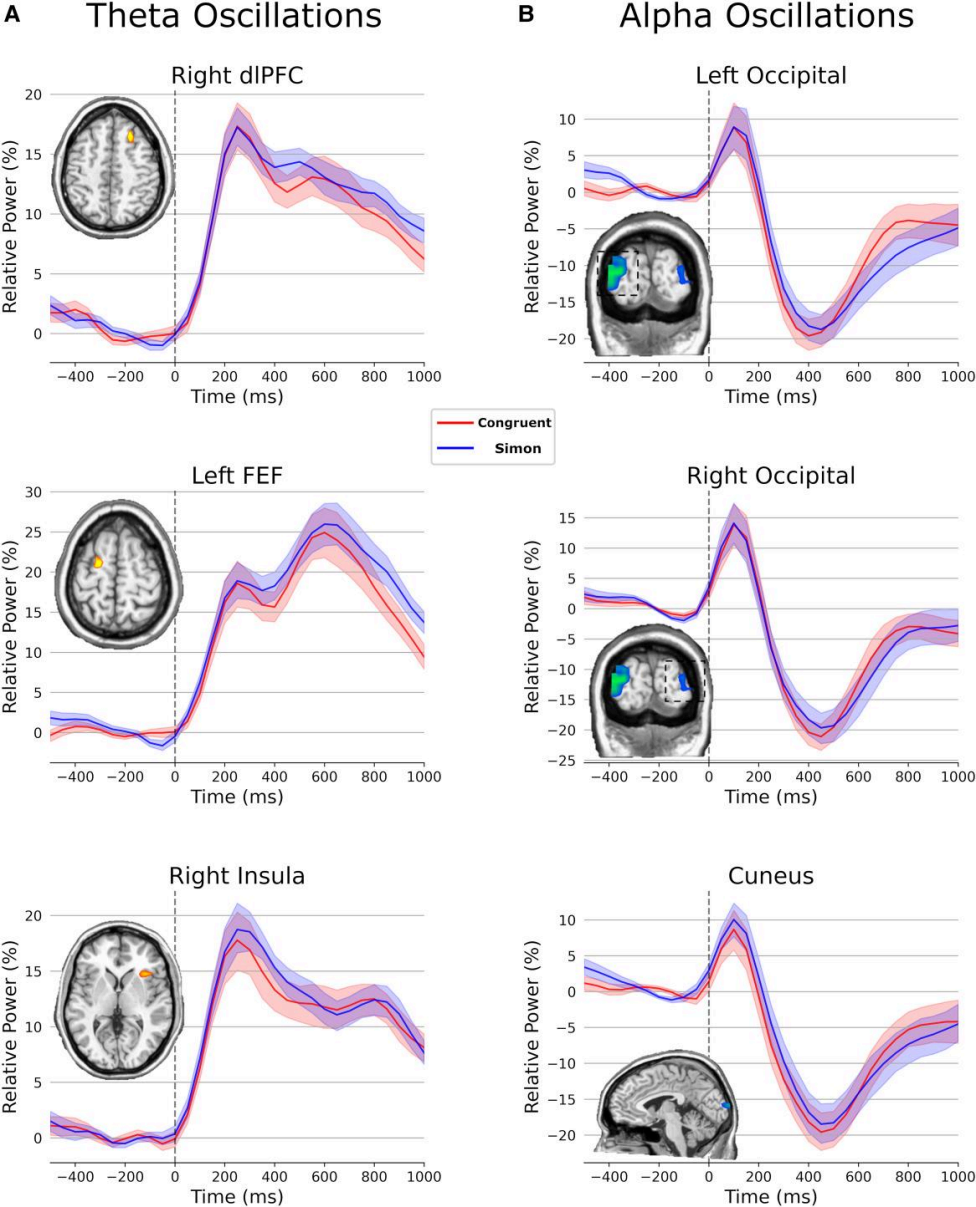
**Theta and alpha task effects.** Time series data were extracted from the peak voxel to visualize the dynamics of each significant cluster in the output maps following one-sample *t*-tests. (**A**) Statistical maps of theta activity collapsing across condition during the 150–500 ms window from 3 to 6 Hz. There were significant theta increases in the right dlPFC, left FEF and right insula (*P* < 10^−12^, corrected). (**B**) Statistical maps of alpha activity collapsing across condition during the 250–600 ms window from 8 to 12 Hz. There were significant alpha decreases in the cuneus and bilateral occipital cortices (all *P*s < 10^−10^, corrected). For each time series plot, the whole-brain statistical map of the task effect, collapsed across both conditions, is shown to the left. Time is denoted on the *x*-axis (0 ms = stimulus onset), while relative power (% change from baseline) is shown on the *y*-axis. Error bars indicate ±1 SEM.

Analysis of the task effects (i.e. across both conditions) showed theta increases across a widespread network that included the left FEF, right dlPFC and right insula (all *P*s < 10^−12^, corrected). The statistical maps and corresponding time series for each cluster are shown in Fig. 3A. For alpha activity, analysis of the task effects showed alpha decreases in the bilateral occipital cortices and the left cuneus during the 250–600 ms time window (all *P*s < 10^−10^, corrected). The time series of each peak and the corresponding functional map is shown in Fig. 3B.

#### Simon effects

**Figure 4:**
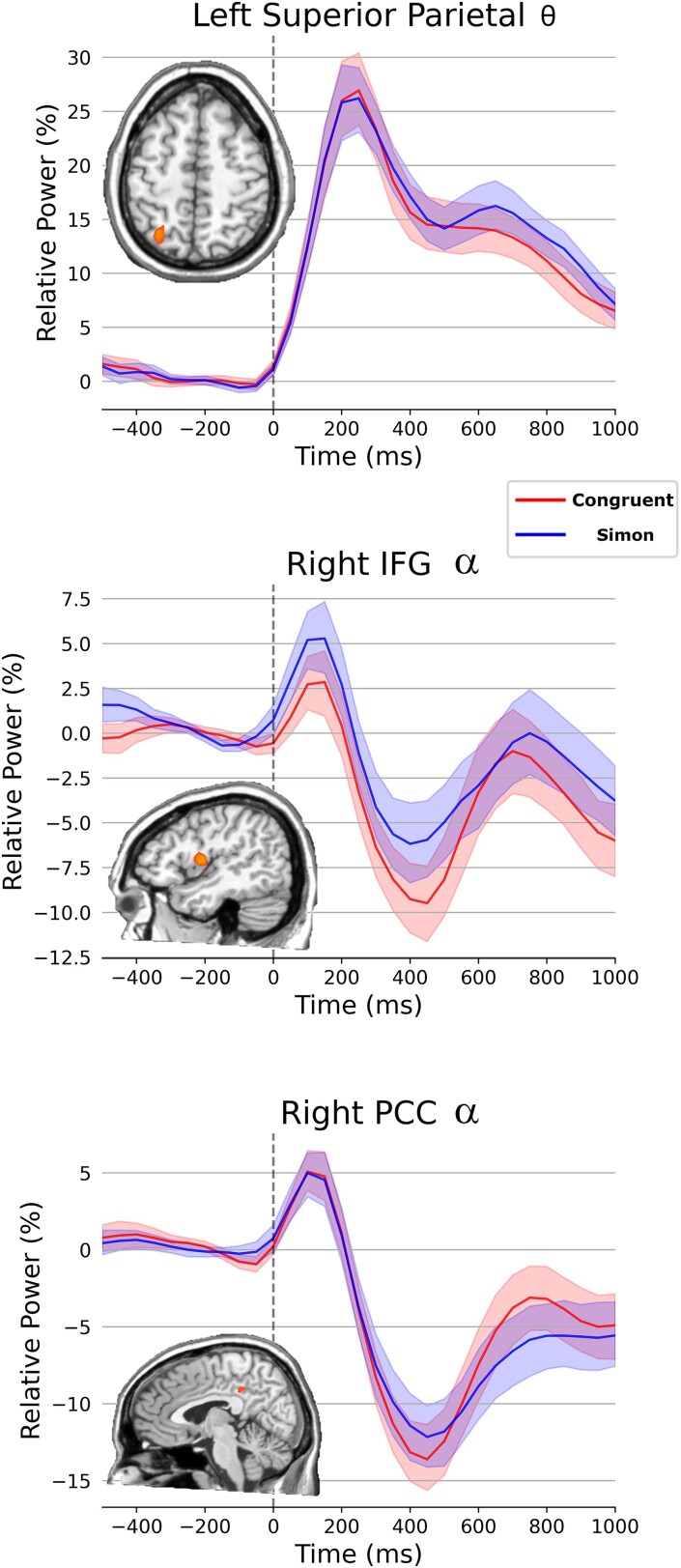
**Whole-brain Simon interference effects.** Time series data were extracted from the peak voxel to visualize the dynamics of each significant cluster in the output maps following paired-sample *t*-tests probing condition-wise effects for theta (150–500 ms) and alpha (250–600 ms) activity. In the theta range, significant condition effects were found in the left superior parietal region (*P* < 0.01, corrected), with the Simon condition being associated with stronger theta responses compared with the congruent condition. Conversely, participants exhibited stronger alpha oscillations (i.e. more negative) during the congruent condition (*P* < 0.01, corrected) in the right inferior frontal region and right PCC. The whole-brain statistical map showing differences between the conditions is shown to the left. Time is denoted on the *x*-axis (0 ms = stimulus onset), while relative power (% change from baseline) is shown on the *y*-axis. Error bars indicate ±1 SEM.

To identify the neural Simon effect, we conducted paired-samples *t*-tests on each oscillatory response map. For the theta time–frequency component, we found significant effects in the left superior parietal region (*P* < 0.01, corrected). For the alpha maps, we detected significant Simon effects in the right inferior frontal gyrus and right posterior cingulate cortex (PCC) (*P*s < 0.01, corrected; Fig. 4). The time series of each peak is also shown in Fig. 4.

## Discussion

In the present study, we probed the spatiotemporal oscillatory dynamics serving visual selective attention performance using advanced source reconstruction of MEG data collected during the modified Simon task in healthy adults. We observed significant oscillatory responses in both the theta (3–6 Hz) and alpha (8–12 Hz) frequency bands, with beamformer imaging indicating a distributed network of brain regions in the frontoparietal attention network that is implicated in visual selective attention and more broadly cognitive control.^54-59^ In the theta frequency range, these regions included the right dlPFC, left FEF and right anterior insula, whereas in the alpha frequency, significant oscillatory responses were found in the bilateral occipital lobes and the cuneus. These regions confirmed our hypothesis that cortical regions strongly implicated in attention networks would be involved in the performance of the Simon task. We also found condition-wise effects (i.e. Simon effect) in the theta frequency in the left superior parietal cortex extending into the intraparietal region. In the alpha frequency, condition-wise effects were identified in the right inferior frontal region and right PCC. Below, we discuss the implications of these findings for understanding visual attention function during the performance of the modified numeric Simon task.

The differential recruitment and patterns of oscillatory activity in the alpha and theta bands may reflect the functional specialization of distinct neural populations pertinent to selective attention processing. For instance in the theta frequency, a distributed network of extrastriate regions that undergo strong increases in neural activity, including the dlPFC, FEF and insula, which are the components of the frontoparietal attention network that have reliably shown activation during attention-demanding tasks.^54-56,60,61^ Oscillatory theta activity has been implicated in the long-distance transmission of stimulus information, which occurs intuitively across the frontoparietal attention network serving selective attention.^62,63^ The dlPFC and FEF have been widely implicated in the top-down modulation of visual selective attention and cognitive control, as well as attention allocation when distracting stimuli are present,^64-67^ while the insula is more commonly associated with facilitating the bottom-up detection of salient events.^68^ Thus, it appears that these regions interact synergistically to achieve the task demands. Oscillatory activity in the alpha range in the bilateral occipital lobes and cuneus has been linked with basic visual processing and components of the striate cortex. These regions have been shown to reliably desynchronize in response to visual stimuli and are involved in the suppression of irrelevant visual information during conflict resolution.^69,70^ These results indicate that these distinct brain regions and oscillatory signatures may serve different functions in selective attention processes during visual selective attention tasks.

Furthermore, we identified neural oscillations in distinct neural populations that differed between the Simon and congruent conditions, indicating regional differences that were specific to visual selective attention. In the theta band, the activity of the superior parietal region was found to be greater in the Simon relative to the congruent condition. The superior parietal region is known to be important in the sustained deployment of selective attention to spatial locations as part of the frontoparietal attention network.^29,57^ Since the superior parietal region is thought to facilitate selective attention, it makes intuitive sense that this region is more active in the Simon than in the congruent condition. In contrast, in the alpha band, the activity of the right inferior frontal region and PCC was greater in congruent trials relative to Simon’s trials. While the anterior cingulate cortex has been implicated in conflict monitoring during task completion, the role of the PCC in selective attention is less understood. The PCC is a central component of the default mode network and is reliably more active at rest than during a task.^71-73^ Thus, greater activity of the PCC in the congruent, or simpler condition, may reflect a decrease in attention towards external stimuli and more internalizing cognition. As for the right inferior frontal region, the stronger alpha oscillations during the congruent condition may reflect increased bottom-up sensory integration, as the inferior frontal region is an interface for the ventral and dorsal attention networks.^4^ However, future studies are warranted to further probe both of these responses.

Our findings are highly compatible with previous EEG spectral studies, which linked increases in theta and alpha power to changes in spatial attention and the suppression of irrelevant visual information.^30-34,69,70^ Our findings extend previous results by harnessing the advantages of MEG, including improved spatial precision to identify and separate cortical sources.^35,36^ In addition, while there have been previous MEG studies that examined visuospatial oscillatory dynamics, there have only been two studies that utilized the classic Simon task to probe visual selective attention in healthy adults.^74,75^ However, both the studies were limited in sample size (*N* < 20) and did not use a data-driven approach to identify statistically significant spectral and temporal windows for beamforming. In addition, Rosenberg *et al*. did not examine spectrally specific differences in neural activity, while van Es *et al*. focused on the response selection (i.e. motor) component of the task. Finally, a third study included a Simon condition in the context of a multisource interference task, but this work mainly focused on the multisource versus single source (Flanker and Simon) interference effects.^23^ Thus, our results contribute to these previous findings by focusing on the oscillatory dynamics of attention networks in a spectrally and temporally specific fashion, with activation in brain regions previously shown to be associated with visual selective attention.^8,9,23,76^ We provide clear evidence that oscillatory activity plays an important role in visual selective attention, and future studies using similar methods will continue to improve our understanding of the cortical dynamics underlying visual selective attention.

Before closing, it is important to note some limitations of this study. For example, this study focused on selective visual attention and future studies could investigate the effects of the modified numeric Simon task on oscillatory activity related to response selection. Second, while we quantified the local dynamics, cognitive interference may impact neural communication at the network level; thus, future studies should focus on dynamic functional connectivity between distinct nodes to further understanding of interregional interactions. Furthermore, we focused our investigation on the spatiotemporal dynamics in healthy young adults; future work could expand these findings by probing the impact of healthy development and psychiatric conditions on such cortical dynamics. Despite these limitations, the current study strongly contributes to our understanding of the neural oscillatory dynamics underlying Simon task performance and the specific impact of Simon interference. In conclusion, we demonstrated robust alpha and theta oscillations in the frontoparietal attention network during stimulus–response resolution, as well as Simon interference effects across distributed brain regions. While visual selective attention has most commonly been examined using the Flanker task, the current findings provide network-specific insights into the oscillatory dynamics serving visual selective attention during stimulus–response resolution in the context of a modified Simon task.

## Supplementary Material

fcad131_Supplementary_Data

## Data Availability

The data used in this article will be made publicly available through the COINS framework at the completion of the study (https://coins.trendscenter.org/).

## Abbreviations

dlPFC =: dorsolateral prefrontal cortex
FEF =: frontal eye fields
MEG =: magnetoencephalography
PCC =: posterior cingulate cortex
