# Supplementary material for: Oscillatory dynamics serving visual selective attention during a Simon task
Source: Brain Commun. 2023 Apr 20;5(3):fcad131. doi: 10.1093/braincomms/fcad131 (PMC10162684; doi:10.1093/braincomms/fcad131)
Supplement: fcad131_Supplementary_Data [file fcad131_supplementary_data.docx]

**Supplementary Materials: Exclusion Criteria**

All participants were screened using a six-item screening form to ensure they met our Inclusion and Exclusion Criteria. Exclusion criteria included:

- Active drug or alcohol abuse/dependence.
- Any major neurological disease such as multiple sclerosis, epilepsy, stroke, Alzheimer’s disease, Mild Cognitive Impairment, Parkinson’s disease, ALS, etc.
- Active brain infection, brain neoplasm, or space-occupying brain lesion.
- Severe psychiatric illness such as schizophrenia, autism, bipolar disorder, major depressive disorder, ADHD, etc.
- History of significant head trauma.
- Current delirium or intoxication.
- Pregnancy.
- General medical conditions: any major medical conditions that would interfere with involvement in the study or may affect CNS function as judged by the investigative team
- The presence of any ferrous metal implant, including orthodonture, which may interfere with the MEG data acquisition and/or be an MRI safety concern.
